# Pilot-scale feasibility study for the stabilization of coal tailings via microbially induced calcite precipitation

**DOI:** 10.1007/s11356-022-22316-1

**Published:** 2022-09-15

**Authors:** Sarah Rodin, Pascale Champagne, Vanessa Mann

**Affiliations:** 1 Department of Civil Engineering, Queen’s University, Kingston, ON K7L 3N6 Canada; 2 Department of Chemistry, Queen’s University, Kingston, ON K7L 3N6 Canada; 3 Centre Eau Terre Et Environnement, Institut de La Recherche Scientifique, Quebec, QC G1K 9A9 Canada

**Keywords:** MICP, Calcium carbonate, Soil stability, Mine waste, Coal

## Abstract

Sustainable long-term solutions to managing tailings storage facilities (TSFs) are integral for mines to operate in a safe and environmentally responsible manner. The long-term storage of subaqueous tailings can pose significant safety, environmental, and economic risks; therefore, alternative containment strategies for maintaining geochemical stability of reactive materials must be explored. In this study, the physical and geochemical stabilization of coal tailings using microbially induced calcite precipitation (MICP) was evaluated at a laboratory pilot scale. Three application techniques simulated commonly used agricultural approaches and equipment that could be deployed for field-scale treatment: spraying on treatment solutions with irrigation sprinklers, mixing tailings and treatment solutions with a rototiller, and distributing treatment solutions via shallow trenches using an excavator ripper. Test cells containing 1.0 × 1.0 × 0.5 m of tailings were treated with ureolytic bacteria (*Sporosarcina pasteur*ii) and cementation solutions composed of urea and calcium chloride for 28 days. Penetrometer tests were performed following incubation to evaluate the extent of cementation. The spray-on application method showed the greatest strength improvement, with in an increase in surface strength of more than 50% for the 28-day testing period. The distribution of treatment solution using trenches was found to be less effective and resulted in greater variability in particle size distribution of treated tailings and would not be recommended for use in the field. The use of rototilling equipment provided a homogenous distribution of treatment solution; however, the disruption to the tailings material was less effective for facilitating effective cementation. Bacterial plate counts of soil samples indicated that *S. pasteurii* cultures remained viable in a tailings environment for 28 days at 18 °C and near-neutral pH. The treatment was also found to stabilize the pH of tailings porewater sampled over the 28-day incubation period, suggesting the potential for the treatment to provide short-term geochemical stability under unsaturated conditions.

## Introduction

One of the most widespread issues faced by the global mining industry is the long-term liabilities associated with the storage of mine tailings in tailings storage facilities (TSFs). Depending on the type of ore-body and associated mineralogy of the surrounding parent rock, tailings may comprise sulfide-bearing minerals, such as pyrite (FeS_2_), which can undergo a series of chemically and microbiologically catalyzed oxidation reactions in the presence of air and water to form ferric iron (Fe^3+^), acidic protons, and sulfate, resulting in increased acidity (Garcı et al. 2005). This process, also known as acid rock drainage (ARD), results in the dissolution and mobilization of metal contaminants (Kefeni et al. 2017). If not properly contained, the seepage of acidic water from a TSF has the potential to contaminate surrounding ecosystems, waterways, and communities, creating health and environmental hazards (Karaca et al. 2018).

Tailings management is especially of concern once a site has been decommissioned, as TSFs and their associated dam structures must be managed until they are no longer a geotechnical hazard, which in some cases may require perpetual maintenance and monitoring (McHaina 2001). For potentially acid-generating (PAG) tailings, measures must be taken to prevent long-term sulfide mineral oxidation and ensure contaminants of concern are immobilized (Johnson 2003). Many ARD prevention strategies have been developed for active operations that have planned for eventual closure, such as filtering, dry-stacking, and desulfurization (Kotsiopoulos and Harrison 2018; Murphy et al. 2012). However, these ex situ management practices are not always practical for abandoned and legacy mine sites. Standard practice often involves sub-aqueous tailings storage, which involves the management of tailings deposits under water in impoundments constructed with earth-filled dams to limit oxygen diffusion (Aps and Aarhus 2001). While this method may be a cost-effective approach for limiting ARD formation in the short term, it can be a significant geotechnical hazard and require on-going maintenance and inspection in the long term. Slope instability, extreme weather events, overtopping, and excessive stress causing liquefaction can all contribute to the failure of a tailings impoundment (Kossoff et al. 2014a). A failure can result in the release of large volumes of tailings and process-affected water, which has the potential to devastate surrounding terrestrial and aquatic ecosystems, and presents a significant environmental and safety hazard (Dimitrova and Yanful 2012; Kossoff et al. 2014b). Dry covers, which use the application of clay minerals or other geosynthetic materials to limit the ingress of oxygen and water, are commonly used as a more stable alternative to sub-aqueous storage; however, these can be expensive for large impoundments and require ongoing maintenance (Tiwary 2001). Therefore, the adoption of alternative long-term storage methods that can improve both geochemical and geotechnical stability are needed.

Bioremediation technologies have been increasingly studied as low-cost remediation strategies for mining effluent (Praharaj and Fortin 2008; Skousen et al. 2017). An emerging technology that could serve as a viable method in the stabilization of TSFs is microbial-induced calcite precipitation (MICP) (Lai et al. 2020; Tamayo-Figueroa et al. 2019; Zhang et al. 2020).

1 $${CO\left(N{H}_{2}\right)}_{2}+2{H}_{2}O\to 2N{{H}_{4}}^{+}+{C{O}_{3}}^{2-}$$

2 $$C{a}^{2+}+ {C{O}_{3}}^{2-}\to CaC{O}_{3}$$

This process involves bacterial species that possess an ureolytic enzymatic pathway, which is responsible for hydrolyzing urea (CO(NH_2_)) into carbonate and ammonium (Eq. 1) (Jain and Arnepalli 2019). In the presence of calcium ions, carbonates precipitate calcite (CaCO_3_) crystals using bacterial cell walls as nucleation sites (Eq. 2) (Ng et al. 2012)*.* This treatment strategy has been applied to a range of applications such as concrete remediation, wind erosion prevention, and landform stabilization (Arpajirakul et al. 2021; Bayat et al. 2021; Pungrasmi et al. 2019).

The precipitation of CaCO_3_ crystals on the surface of sediment facilitates binding at the contact points between tailings particles and reduces the size of the pore spaces, which can improve the mechanical strength of the soil matrix and reduce permeability (Gui, et al. 2018; Harkes et al. 2010; Punnoi et al. 2021). This treatment could be advantageous for tailings attenuation, as the formation of a cemented “biocrust” in the upper layer of tailings would reduce water and oxygen ingress, thus limiting the exposure of stored tailings to the weathering processes that control ARD. This process could also serve as a means to sequester metal species that are prevalent in mine waste, by forming insoluble metal complexes, thus reducing the concentration of soluble heavy metals (Yang et al. 2016). The bioaugmentation of ureolytic species to perform in situ cementation could offer the benefits of traditional clay soil covers for permeability reduction, as well as alkalinity generation that is traditionally supplied by lime addition (Chang et al. 2000). The use of this type of in situ treatment would be especially beneficial to a legacy site where material rehandling is often impractical or cost-prohibitive. Bench-scale studies have been proven effective for the cementation of mine tailings using injection, percolation, and submerge methods to apply treatment solutions (Oualha et al. 2020; Zamani et al. 2019). However, few pilot studies have been conducted on a viable way to apply cementation media to tailings material; therefore, further developments of scaled-up treatment processes are required for large-scale application (Cheng and Shahin 2016; Zhu and Dittrich 2016).

The Victoria Junction Tailings Basin (VJTB) is a legacy TSF in Cape Breton, Nova Scotia, Canada, currently in a state of monitoring and maintenance managed by Public Works and Government Services Canada (PWGSC). The VJTB contains approximately 1,000,000 m^3^ of acid-generating coal tailings that are held beneath a water cover and retained by an earth-filled dam structure. The dam has been in place since the 1980s, and is managed in accordance with the Canada Dam Safety Guidelines. The current closure strategy for the VJTB is to maintain the water cover in perpetuity; however, PWGSC wishes to identify a feasible, reliable alternative to stabilize the tailings material without the requirement of water cover or water treatment and subsequently decommission the dam.

The purpose of this study is to evaluate the feasibility of the MICP process for the stabilization of coal tailings at the laboratory pilot-scale. To further evaluate the scalability of this treatment, suitable application strategies for treatment solutions were selected based on existing techniques used in agricultural practices of a similar scale. The three potential strategies examined in this study are as follows: spraying on treatment solutions with irrigation sprinklers, mixing tailings and treatment solution with tilling equipment, and distributing treatment solution via shallow trenches.

## Experimental approach

### Tailings specimens

The tailings material was collected from the VJTB site which consists of fine to medium sand with trace to some silt and clay. Soil moisture conditions were highly variable, with a mean moisture content of 30%. Results from sieve analyses and moisture contents of seven different samples taken from the site are provided in Table 1.

**Table 1:** Soil texture analysis and measured moisture contents of seven samples taken from the VJTB site

	S-1	S-2	S-3	S-4	S-5	S-6	S-7	Mean	SD
Sand (%)	89.0	91.0	85.0	56.0	59.0	65.3	59.0	69.2	15.6
Silt (%)	6.0	5.0	8.0	26.0	24.0	19.3	23.0	17.6	9.21
Clay (%)	5.0	4.00	7.0	18.0	17.0	15.3	18.0	13.2	6.40
Moisture (%)	22.1	9.6	14.0	44.1	43.7	33.1	35.8	30.0	13.90

Mineralogical analysis completed on tailings samples indicated that pyrite comprises approximately 10% of the tailings mass. The crystalline fractions consisted mainly of silicate and aluminosilicate minerals, including kaolinite, illite, mica (i.e., muscovite, biotite), and quartz. Calcite comprises an estimated 2 to 3% of the tailings mass.

### Pilot cell set-up

**Fig. 1:**
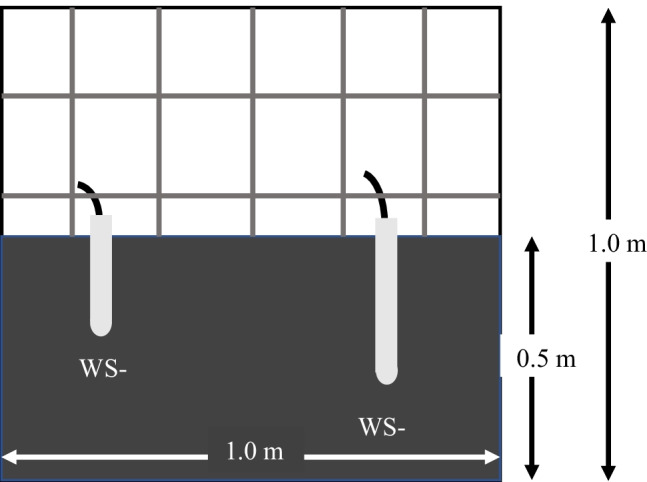
Illustration of pilot cell cross section with coal tailings and porewater samplers

Each pilot-scale test was carried out in a 1-m^3^ intermediate bulk container (IBC), which was subsequently filled with tailings to a depth of 0.5 m (0.5 m^3^ total test volume). To ensure homogeny between tests, tailings of different material classifications were added to each test cell in equal proportions and mixed thoroughly. Prior to treatment application, tailings were kept under a 5-cm water cap using water collected from the tailings basin to maintain material saturation. Two slim-tube water samplers (Soilmoisture Equipment Corp.) were installed in each cell to allow for pore water samples to be collected. For each cell, one 30-cm-long sampler (WS-A) was placed at the north-west corner and a 46-cm-long sampler (WS-B) was placed at the southeast corner. To establish conditions similar to a water cap removal, tailings remained saturated upon application (Fig. 1).

### Bacteria and growth conditions

*Sporosarcina pasteurii* (ATCC 11,859) was selected for this study as it has been widely studied for MICP applications and it is a robust organism with a high urease activity (Williams et al. 2016). *S. pasteurii* was cultivated in yeast extract medium, composed of 10.75 g/L Tris base (C_4_H_11_NO_3_), 2.0 M HCl to adjust pH to 9.0, 20 g/L yeast extract, and 10 g/L ammonium sulfate. Media was sterilized in 10.0 L batches by autoclaving at 121 °C for 2 h. Approximately 200 mL of inoculum was cultivated in a shaker incubator at 31.0 °C and 150 rpm. Optical density measurements at 600 nm (OD_600_) were taken twice daily to monitor growth. When the culture reached an optical density of 1.0–1.5, 80 mL was transferred to 2 × 1.5 L sterile bench top reactors, where monitoring of growth continued. Samples were periodically taken for streaking on a yeast extract-agar medium to assess the morphology of the pure cultures. When cultures reached an optical density of 1.0, 500 mL from each bench top reactor was used to inoculate two 27 L batch reactors. After a 5-day growth period, bacterial plate counts on yeast extract-agar plates indicated an average of 5.82 $\times$ 10^8^ CFU/mL. Growth media was separated from the resulting cell mass by centrifugation at 2000 rpm for 15 min and resuspended in the prepared urea solution.

### Treatment solutions

Two treatment solutions were prepared, one supplying urea and another supplying calcium chloride (CaCl_2_). To prepare the urea solution, 3.0 g/L nutrient broth, 2.12 g/L sodium bicarbonate, and 10.0 g/L of ammonium chloride were dissolved in deionized water. Filter-sterilized urea was then added to the sterile solution to achieve a urea concentration of 0.33 M. Grown *S. pasteurii* cultures were centrifuged at 2000 rpm for 15 min, and resultant cell masses were resuspended in the urea solution to form a urea-bacteria suspension (UBS). A 1.0 M CaCl_2_ solution (CS) was prepared by dissolving 280 g CaCl_2_.2H_2_O in 2.0 L of deionized water and autoclaving at 121 °C for 1 h to sterilize. Treatment solutions were applied to each pilot cell separately, with a 3:2 molar ratio of urea to CaCl_2_ (Wong 2015).

### Treatment application methods

For each treatment, which was completed in situ, 8.0 L of UBS was applied to the tailings surface, along with 4.0 L of CS. A temperature of 18 °C was maintained for the duration of the test following application, as this is the average seasonal temperature at the VJTB site. A summary of test cell conditions is shown in Table 2.

**Table 2:** Summary table of treatment conditions for the four pilot cells evaluated

Cell	Description of application
Control	No treatment
Spray	UBS and CS sprayed-on
Till	UBS and CS mixed into tailings with roto-tiller
Trench	UBS and CS dispensed in shallow trench

#### Control

The control pilot cell, which was monitored for 28 days with no addition of either treatment solution.

#### Spray irrigation (Spray)

For simulating a spray-on application, 4.0 L commercially available pesticide sprayers were used, one containing UBS, and another containing CS. Prior to use, each sprayer was flushed with 70% ethanol and DI water to disinfect. Immediately after being filled, solutions were applied to the surface of the pilot cell at a rate of 150 mL/min. To ensure adequate contact between treatment solutions and the material surface, this application was completed in 2 sequential applications of equal volume, 4 days apart.

#### Rotary cultivation (Till)

A rotary cultivator was used to simulate soil tilling equipment. Both treatment solutions were poured across the surface of the pilot cell at an approximate rate of 0.25 L/s, after which, the upper 15 cm of soil was tilled in 1.0 m × 0.3 m sections to allow for adequate mixing between tailings and treatment solutions.

#### Shallow trench formation (Trench)

A spade shovel was used to simulate the actions of a ripper excavator. The spade was inserted approximately 15 cm below the tailings surface and was pulled across the length of the pilot cell to form a shallow trench. Respective treatment solutions were subsequently poured into the newly formed trench at a rate of approximately 0.5 L/s.

### Monitoring methods

#### Surface strength

Strength testing was performed to evaluate the effect of cementation on the tailings surface using a pocket penetrometer (AMS Inc 59,032). Pressure was applied to a loading piston until a depth of 6 mm was reached or the material failed, and the corresponding strength, measured in kg/cm^2^, was recorded in kilopascals. The surface of each pilot cell was divided into 24 equal sections for localized strength measurements, which were conducted in triplicate. Based on the material strength requirements determined for the test site to proceed with closure activities, a compressive strength of 100 kPa was targeted.

#### Particle size distribution

Tailings samples were collected from each pilot cell after its test duration and were taken at 10-cm increments beginning from surface-level to 50 cm below the surface. Particle size distributions were determined using sieve and hydrometer analysis (ASTM D422) by AGAT Laboratories (ASTM International 1998).

#### Mineralogical and geochemical analysis

At the end of the test period, treated samples were collected at the material surface, as well as at a depth of 0.3 m below the surface in order to assess the permeation of the treatment solution below the cell surface. X-ray diffraction (XRD) analysis was completed by AGAT Laboratories to quantify the content of CaCO_3_ minerals in sampled tailings. Representative samples were micro ionized with a planetary ball mill and analyzed with X’pert PRO X-ray diffractometer (Cu*K*α radiation) with an angular range from 4 to 75˚2θ. Mineralogy was interpreted using ICDD PDF-4 mineral database and Rietveld Refinement using TOPAS software. Acid–base accounting (ABA) was completed using the Sobek method to determine acid-producing potential (APP), neutralizing potential (NP), and sulfur content of tailings samples. Shake flask extraction (SFE) tests were also completed on sampled tailings to analyze the dissolved compounds in the leachate of sampled tailings. Samples were leached in deionized water for 18 h using a 3:1 liquid–solid ratio. Resultant leachate was analyzed for water quality parameters and dissolved metals. ABA and SFE tests were completed by RPC Engineering.

#### Colony-forming units test

Agar media was comprised of 20 g/L yeast extract, 10 g/L ammonium sulfate, and 20 g/L nutrient agar. Two soil samples were collected from each pilot cell, one at 14 days after application, and one after 28 days. Forty grams of soil was suspended in 40 mL of deionized water and left to stand for 30 min. Then, 0.5 mL of soil suspension was pipetted into 4.5 mL of sterile deionized water, after which, a serial dilution was completed from 10^−1^ to 10^−6^ mL. Then, 200 μL of each dilution was spread over agar plates and incubated 31 °C for 36 h. Plate counts for each soil sample were performed in duplicate (Bhaduri et al. 2016).

#### Water quality

Porewater samples were collected from each pilot cell at depths of 0.3 m and 0.46 m using two slim-tube water samplers. The pH of samples was determined using a pH probe (Fischer Accumet 13–620-31C) and meter (Accumet XL60). Ammonia nitrogen (NH_3_-N) concentrations in pore water samples were measured using a Hach DR2800 spectrophotometer (Hach Ammonia TNT plus Vial Test). Water samples with concentrations exceeding the detection limit for this test were diluted as necessary. Porewater pH measurements were taken every 4 days and NH_3_-N measurements every 7 days.

### Statistical analysis

All statistical analysis of results was completed using SPSS software. To evaluate compressive strength between test cells, a one-way ANOVA test was completed, and means were compared using a Tamhane’s T2 test (*p* = 0.05). Mean grain sizes for particle size distribution data were calculated using the Folk and Ward method (Folk and Ward 1957). A log_10_ transform was used to normalize results of bacterial plate counts (Corry et al. 2007), and a one-way ANOVA and Tamhane’s T2 test were used to determine any statistically significant differences between application methods. A one-tailed Student’s *T* test ($\alpha$ = 0.05) was also completed to determine whether observed changes in CFU counts were statistically significant between 14 and 28 days. Due to the scale of pilot experiments, biological replication of test conditions could not be completed.

## Results and discussion

### Effects of MICP on compressive strength

**Fig. 2:**
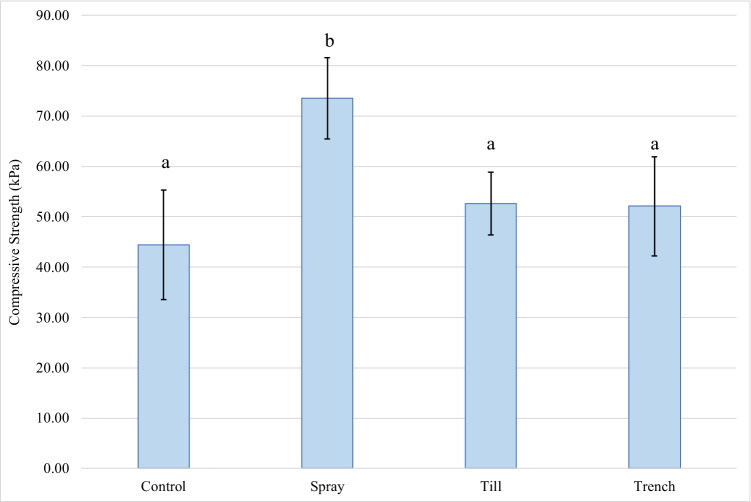
Mean surface strength (kPa) for Control, Spray, Till, and Trench tests completed 28 days after initial treatment application at 18 °C. (*n* = 24). Letters “a” and “b” indicate sample means that were significantly different from one another; sample means with the same letter do not have statistically significant differences (*p* = 0.05)

Surface strength measurement of tailings in each pilot cell was performed 28 days after treatment to assess the mechanical improvements of the material resulting from MICP. A comparison of average material strength between test cells is shown in Fig. 2.

The average surface strength was found to be the lowest in the Control, which was measured as 45.46 kPa. The Spray treatment yielded the highest average surface strength of 73.55 kPa, with some regions of its surface measuring as high as 122 kPa; however, variations in strength measurements were still observed in this cell. The Till treatment exhibited an average surface strength of 52.61 kPa, an approximate increase of 15% from Control. Similarly, the average surface strength of the Trench treatment was 52.10 kPa. Based on the one-way ANOVA completed, Spray was found to be the only MICP-treated cell in which a significant increase in material surface strength was observed compared to the Control.

**Fig. 3:**
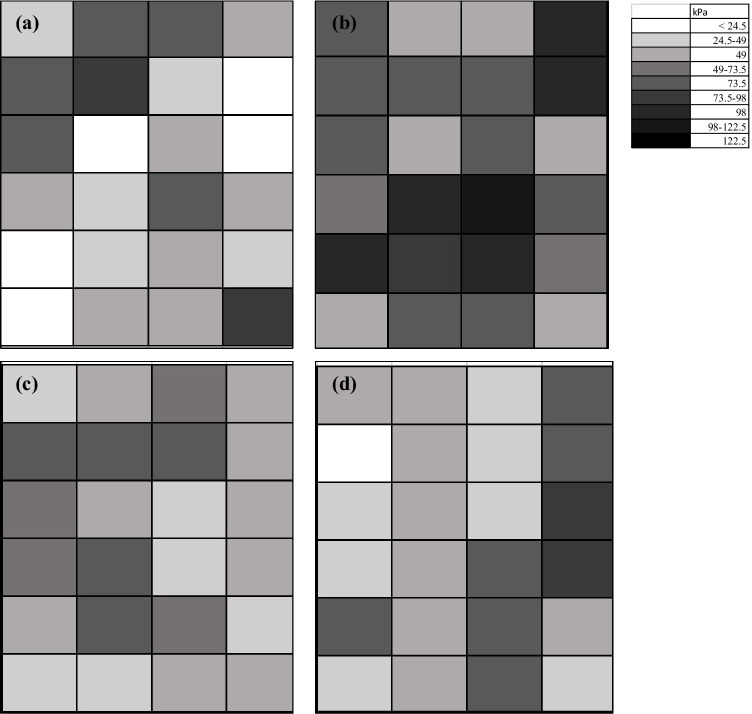
Measured penetrometer strength (kg/cm^2^) across surface of pilot cells (**a**) Control, (**b**) Spray, (**c**) Till, (**d**) Trench

As a result of the variations in strength measurements and the large surface area of each pilot cell, a graded map of reported measurements was also created to illustrate localized improvements in material strength (Fig. 3). The Control exhibited the widest variation in strength measurements, with a standard deviation of 24.1 kPa. Till exhibited the most homogeneity across surface with a standard deviation of 14.7 kPa. In comparison, the spray-on cell had a standard deviation of 19.13 kPa. The strength measurements in Trench showed a larger variation than the Control, with a standard deviation of 23.21 kPa. In the region of the cell in which the initial trench was cut, where higher concentrations of UBS and CS solutions were assumed to be present, the average surface strength was 49.03 $\pm$ 18.53 kPa.

Improvements in material strength were considered a key metric in evaluating the efficacy of different MICP application methods (Ng et al. 2012). Results indicated Spray to be the only application method to yield a significant increase in material strength. This was consistent with previous studies that have successfully used a non-intrusive surface application to distribute treatment solutions within a soil sample (Cheng and Cord-Ruwisch 2012; Hoang et al. 2019). This method showed the least disturbance to the material, allowing the treatment solution to permeate into the pore space of the sediment, thus leading to the nucleation of CaCO_3_ crystals between soil and rock particles. Additionally, the saturated state of the material upon initial application may have allowed the substrates from treatment solutions to be mobilized by the tailings pore water via diffusion. In contrast, both Till and Trench application methods were more disruptive to the tailings material, which likely impacted the extent of cementation. In the case of the Till treatment, the use of tilling equipment that effectively mixed the tailings with the treatment solutions provided the most homogenous result; however, significant improvements in strength were not observed. It is believed that the treatment application caused soil disturbances that have the potential to impact the stress profile of the soil being evaluated (Mujah et al. 2017).

**Fig. 4:**
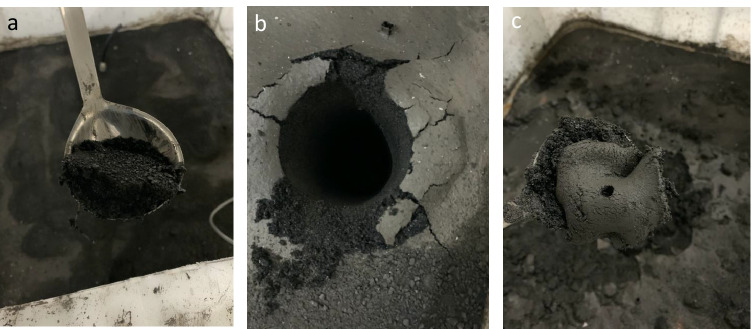
Tailings surface material from pilot cells Spray (**a**), Till (**b**), Trench (**c**) after 28 days at 18 °C

**Fig. 5:**
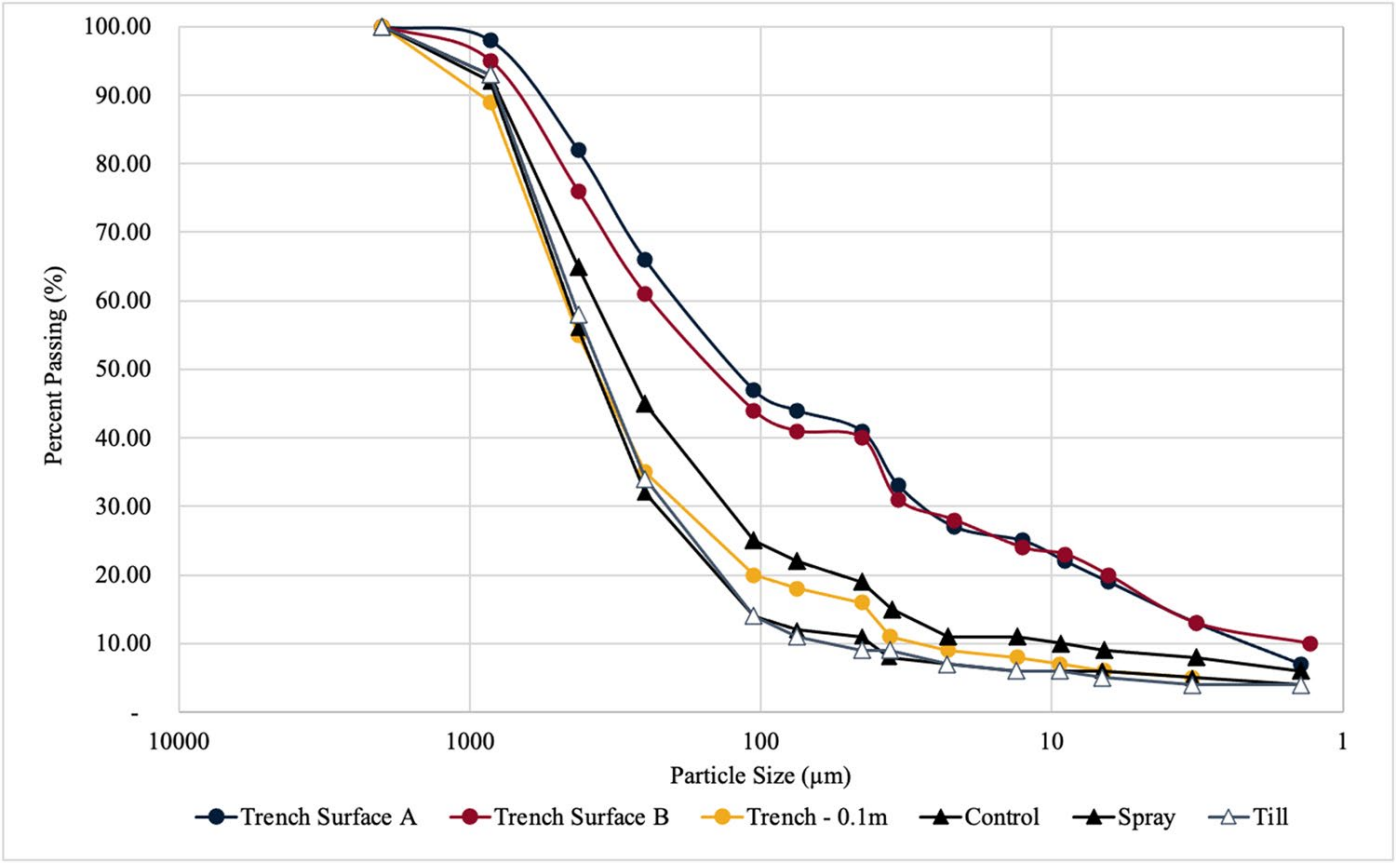
Particle size distributions for VJTB tailings sampled from the Control, Spray, Till, and Trench cell 28 days after initial treatment. “Trench Surface A” and “Trench Surface B” were sampled from the surface of the shallow trench made to distribute treatment solutions; “Trench—0.1 m” was sampled 0.1 m below the surface of the shallow trench

From visual observation, the surface texture of each pilot cell varied considerably (Fig. 4). The surface of tailings sampled from Spray remained intact upon excavation, indicating that a cemented crust formed over the test duration. Till and Trench exhibited a film-like surface that was lighter in color and appeared more fine-grained than the material underneath. Within the Trench cell, it is suspected that the addition of treatment solution and mechanical agitation caused the segregation of granular and fine-grained tailings, which has been observed in soil-freshwater mixtures (Ganesalingam et al. 2013). This can be supported by particle size data for Trench, as the mean grain size of the tailings surface samples was found to be 85–115 m$\upmu$, while the mean grain size 0.1 m below the surface was 307.1 m$\upmu$ (Fig. 5).

It is also noted that tailings samples from Till and Trench had elevated moisture levels compared to material sampled from Control and Spray (Table 3). Soil moisture influences soil engineering properties, which may have contributed to the lower strength observed in the Till and Trench cells. Silt and clay particles exhibit effective moisture retention, and the establishment of fines at the material surface in the Trench cell may have inhibited pore water evaporation in the Trench cell (De Kretser et al. 1997; Guo et al. 2018). However, despite similar particle size distributions and minerology, the tailings in the Till cell had noticeably a higher soil moisture content 28 days after treatment compared to the Spray cell. The physical appearance of the Till cell suggests this treatment application technique may have facilitated soil crusting, whereby fine particles within sandy soil form a thin compacted layer over top of more course-grained underlying particles, resulting in the clogging of pore spaces at the material surface and reduced permeability (Gallardo-Carrera et al. 2007). This has been observed in some tilling applications, and therefore it is possible that any improvements to soil strength made by MICP were likely reduced by alterations to soil properties from respective application strategies (Ndiaye et al. 2005). Soil crusting has been shown to occur through both biotic and abiotic means, and it is unclear the extent of which *S. pasteurii* may have contributed to this process. Given the importance of soil strength to furthering the closure activities at the study site, the Till and Trench application methods are not ideal for field applications. This result also highlights the importance of soil properties on the success of MICP, as the soil conditions created from the Till and Trench treatment were insufficient to see incremental gains in structural soil properties.

**Table 3:** Moisture content of tailings samples collected from Control, Spray, Till, and Trench cells at surface-level (0 m) and 0.3 m below the surface. All samples were collected 28 days after treatment at 18 °C

Moisture (%)	Control	Spray	Till	Trench
0 m	9.9	11.4	88.5	80.9
0.3 m	23.9	18.7	83.0	83.1

A previous study reported that under 100% saturation, the site of calcite crystal formation occurs in pore spaces or sediment surfaces, whereas as 20% saturation, crystal formation occurs at sediment junctions, which are optimal for improving the mechanical properties of soil (Cheng et al. 2013). Therefore, it is possible that the high-moisture environment maintained in Till and Trench over the 28-day treatment period did not facilitate effective crust formation that would improve material strength. It is noted that the relationship between EPS production and carbonate precipitation has been studied, but few studies have looked at the combined influence of these two factors on the mechanical properties of soil (Ercole et al. 2007; Or et al. 2007).

### Effects of MICP on soil geochemistry

**Fig. 6:**
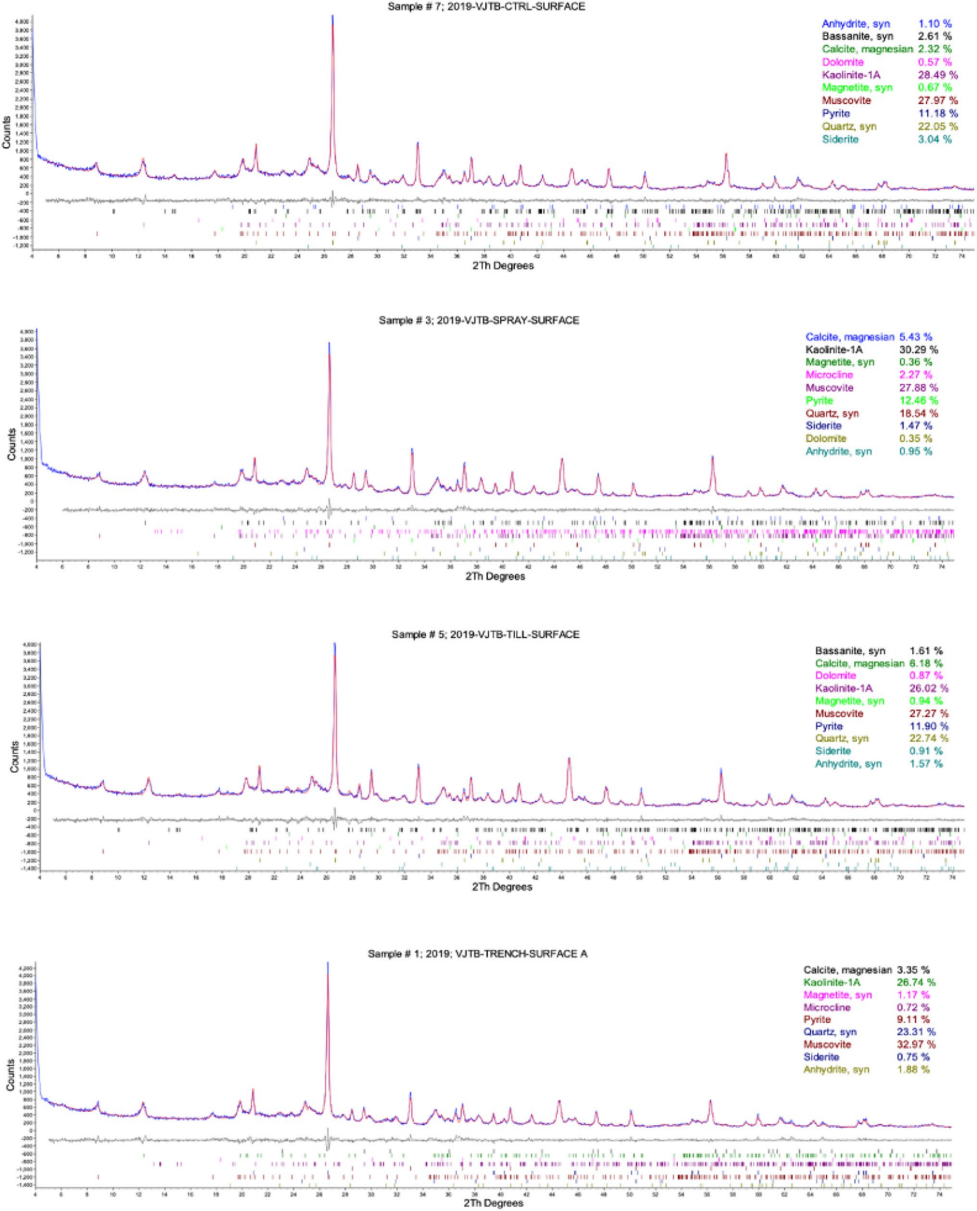
XRD spectra of tailings samples collected from pilot cells (**a**) Control, (**b**) Spray, (**c**) Till, (**d**) Trench

**Fig. 7:**
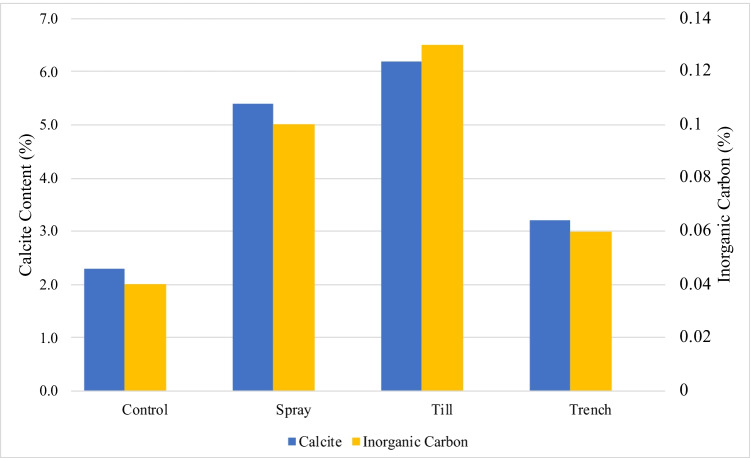
Calcium carbonate (CaCO_3_) content (weight %) and inorganic carbon content (weight %) measured in tailings sampled at the surface of Control, Spray, Till, and Trench pilot cells

Surface samples collected from each pilot cell after 28 days were analyzed to measure calcite (CaCO_3_) content and soil inorganic carbon content, or the measure of carbonate compounds within soil. The results of XRD analysis completed to quantify CaCO_3_ minerals are presented in Fig. 6. Measurements indicated a CaCO_3_ content of 5.4% and 6.2% in Spray and Till after their respective treatments. These were observed increases relative to the Control, which was found to have a CaCO_3_ content of 2.3%. A CaCO_3_ content of 3.3% was recorded for the Trench, which was lower than that observed for the other treated cells. A strong positive correlation was observed between measured CaCO_3_ content and inorganic carbon, indicating that inorganic carbon can also be used as a strong indicator for CaCO_3_ precipitation (Fig. 7).

Of the three treatment applications, Till was found to have the greatest increase in CaCO_3_ and inorganic carbon. This increase suggests the mixing of the tailings material with UBS and CS provided acceptable environmental conditions for *S. pasteurii* to initiate CaCO_3_ formation. Calcite content has previously been found to correlate strongly to soil strength parameters such as shear and compressive strength (Choi et al. 2020). However, this correlation was not observed in the Till cell. This further supports the notion that a rototilling application is not suitable for tailings stabilization as precipitated calcite was not adequately utilized for the cementation of soil particles. Based on both measured calcite and inorganic carbon, Trench was found to have relatively small improvements in precipitated calcite, suggesting the conditions of this treatment application method did not facilitate sufficient calcite precipitation compared to the Spray and Till methods. It is possible that the segregation of fine particles at the surface of the Trench cell could have impacted MICP, as small pore spaces may restrict bacterial movement and the transport of nutrients (Rebata-Landa and Santamarina 2006; Wang and Nackenhorst 2020).

Acid–base accounting completed on samples from each pilot cell provided insight into the geochemical stability of treated tailings (Table 4). Results indicated that a 28-day treatment may not be sufficient to improve the net neutralizing potential of the material. However, analysis of sulfur species showed that all treatments had lower sulfate levels relative to the Control, suggesting the treatment contributed to minimizing the weathering of pyrite (Blowes and Jambor 1990). It should be noted that longer-term kinetic tests would be necessary to understand the effect of this treatment on geochemical weathering.

**Table 4:** Measured acidification potential (AP), neutralizing potential (NP), and percent sulfate of total sulfur for Control, Spray, Till, Trench

	Control	Spray	Till	Trench
AP (kg CaCO_3_/tonne)	104.31	110.34	116.34	107.1
NP (kg CaCO_3_/tonne)	17.23	23.73	29.99	18.2
SO_4_/Total Sulfur (%)	9.54	6.09	4.05	5.83

### Viability of bacterial colonies

**Fig. 8:**
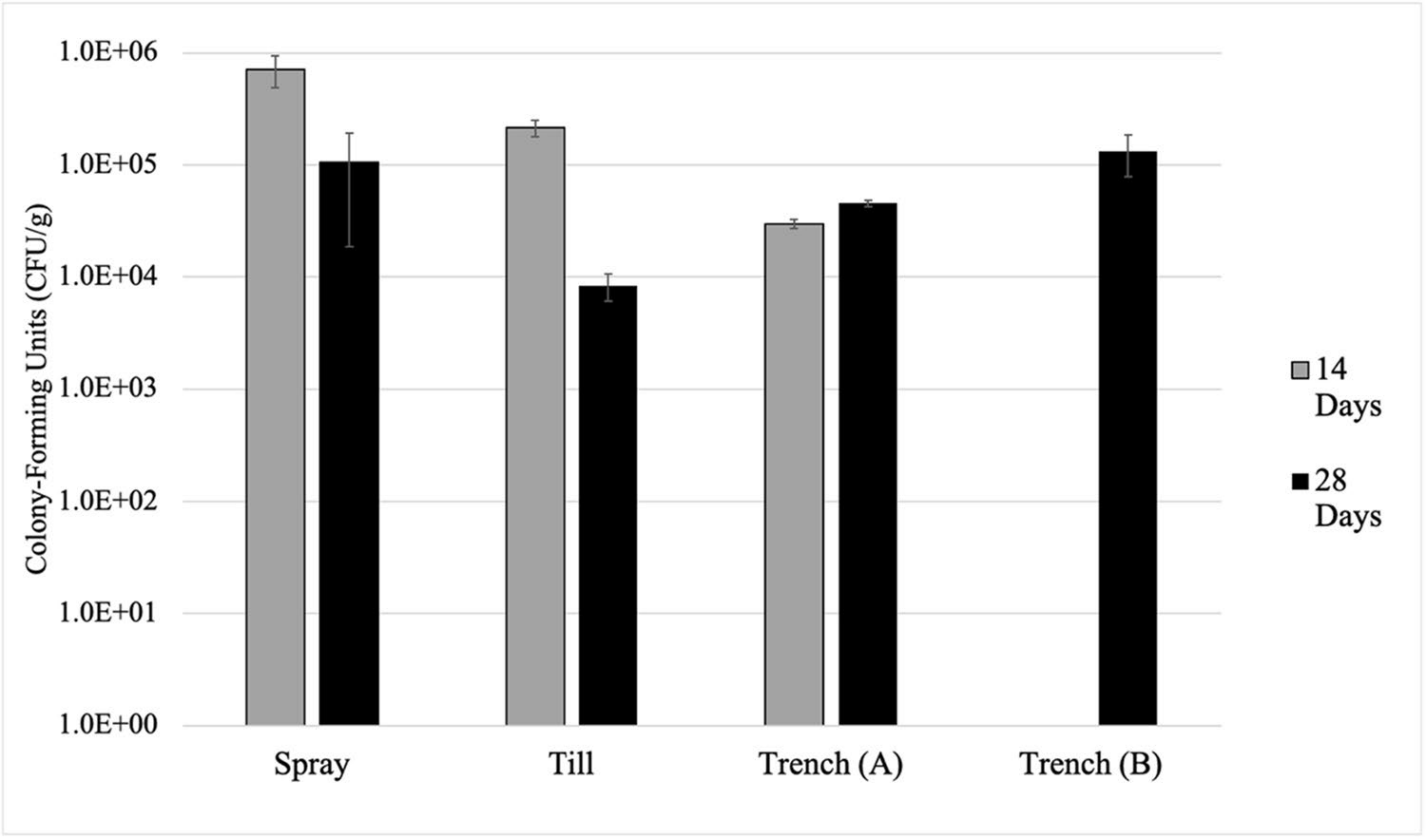
Measured colony-forming units per gram surface tailings (CFU/g) collected from Spray, Till, Trench treatments 14 days and 28 days initial treatment application (*n* = 2). Measured CFU/g from different regions of Trench denoted by “A” and “B”

Bacterial plate counts completed on surface tailings of Control, Spray, Till, and Trench cells 14 and 28 days after initial treatment provided insight into changes to the viability of bacterial populations over the period of testing. Colony-forming units (CFUs) measured from Control samples were all found to be below the range considered statistically significant (30–300 CFU); therefore, these results were not reported (Sanders 2012). A summary of bacterial plate counts is shown below in Fig. 8. It was found that the decrease in viable bacteria in the Spray, Till, and Trench cells from 14 to 28 days was statistically significant; however, there were no statistically significant differences in CFUs between Spray, Till, and Trench treatments.

Higher concentrations of CFUs were measured in Spray, Till, and Trench relative to the Control both 14 days and 28 days after treatment. Spray exhibited the highest number of CFUs, with 7.11 × 10^5^ (± 2.25 × 10^5^) CFU/g found in tailings sampled 14 days after treatment, and 1.05 × 10^5^ (± 8.63 × 10^4^) CFU/g from samples taken 28 days after treatment. Till exhibited the highest observed decrease in CFUs between sampling periods of the three treated cells with 2.10 $\times$ 10^5^ (± 3.68 × 10^4^) CFU/g after 14 days, and 8.4 $\times$ 10^3^ (± 2.26 $\times$ 10^3^) CFU/g after 28 days. Trench showed relatively stable concentrations of viable cells. Tailings samples taken from the trenched region were found to have a colony count of 3.0 $\times$ 10^4^ (± 2.83 × 10^3^) CFU/g after 14 days, and 4.54 $\times$ 10^4^ (± 3.11 × 10^3^) CFU/g after 28 days. Some of the decreases in cell counts could be attributed to the depletion of substrates utilized by bacterial cultures over the 28-day testing period (Mugwar and Harbottle 2016). Due to the suspected uneven distribution of treatment solution using the Trench method, additional samples were collected from a different location of the shallow trench (region B) after 28 days. The tailings taken from trench region were found to have a concentration of 1.31 × 10^5^ (± 5.32 × 10^4^) CFU/g at 28 days, and while this was higher than the concentration initially measured within the shallow trench (region A), the difference was not found to be statistically significant.

The plate counts are indicative of the total amount viable colonies present in each test cell; however, they do not indicate the composition of native microbial populations versus *S. pasteurii* cultures. To account for the possible biostimulation of other native species, a bench-scale testing was completed on VJTB tailings samples, in which treatment solutions were applied both with and without *S. pasteurii*. It was found that after a 28-day period, samples treated with *S. pasteurii* had an average concentration of 3.62 $\times$ 10^5^ CFU/g, compared to nutrient-enriched samples, which had a concentration of 2.05 $\times$ 10^5^ CFU/g. Control samples did not exhibit significant colony growth to enable CFU determination. This would suggest that *S. pasteurii* can remain viable in the tailings environment; however, native species may also utilize treatment solutions as substrate, which could result in competition for nutrients. It is not known whether native species in these tailings possess enzyme pathways to facilitate CaCO_3_ precipitation; therefore, a more in-depth microbiological analysis of bacterial species within the tailings and their interactions would be suggested in future work to gain a better understanding of its impact on MICP.

### Effects of MICP on water quality parameters

**Fig. 9:**
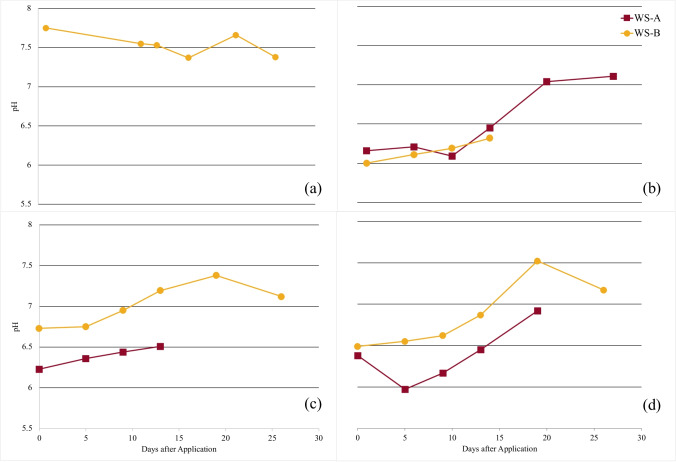
Porewater pH measurements taken over test period from 0.3 m (WS-A) and 0.5 m (WS-B) for Control (**a**), Spray (**b**), Till (**c**), and Trench (**d**)

Porewater pH was used to monitor the progression of MICP in each cell, as well as potential ARD formation. The recorded pore water pH for each test cell is shown in Fig. 9. The initial porewater pH of Spray, Till, and Trench was lower than the Control which was likely due to the need for additional site water cap (pH = 6.3) to saturate the tailings in these pilot cells.

The analysis of porewater from WS-B in the Control indicated no decline in pH approximately 0.5 m below surface level during testing, providing a positive outlook on the short-term geochemical stability of this material under neutral conditions. The recorded pH of the porewater collected from WS-B in MICP-treated cells steadily increased over the 28-day period, with porewater samples stabilizing approximately 21 days after treatment, and reaching a final pH of 7.0–7.5. A similar observation was made for samples collected from WS-A. Due to reduction in moisture from evaporation, some WS-A samplers could not collect a sufficient volume of pore water for analysis. Results from shake flasks extractions completed on tailings from a 0.3-m depth 28 days after treatment, were found to be approximately 7.6 in Spray, Till, and Trench treatments, suggesting further improvements in water quality occured over the remainder of the test period.

**Fig. 10:**
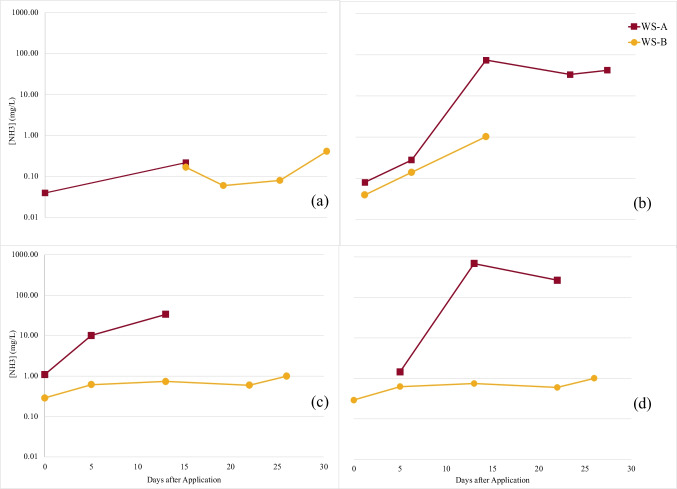
Porewater NH_3_-N measurements taken over test period from a depth of 0.3 m (WS-A) and 0.5 m (WS-B) for pilot cells (**a**) Control, (**b**) Spray, (**c**) Till, (**d**) Trench

The measurement of ammonia (NH_3_) is also commonly used to indicate the extent to which MICP has occurred. NH_3_ concentrations measured from pore water samples from each cell are shown in Fig. 10. Marginal increases in NH_3_ were also observed in WS-B over the 28-day period, which can likely be attributed to the diffusion of NH_3_ through the saturated pilot cells to depths of 0.46 m below the surface.

NH_3_ measured in the Control remained low throughout the test duration, averaging 0.163 $\pm$ 0.14 mg/L. In treated test cells, NH_3_ concentrations were found to be slightly above levels measured in the Control after initial application. After 14 days, an observable increase in the concentration of NH_3_ occurred in each of the three treated cells. The increase was more visible in samples taken from WS-A, as it was situated closer to the tailings surface in each cell, indicating urea hydrolysis was more prevalent in the upper layer of tailings. Measurements taken from WS-A over the remainder of the test period showed NH_3_ concentrations stabilizing or decreasing slightly. Tailings leachate analyzed by shake flask extraction showed similar results to the porewater samples. NH_3_ measured from the tailings leachate of the Control samples remained low, as shown in Table 5. The concentration of NH_3_ in the leachate of Spray, Till, and Trench was found to be higher than the Control. Notably, the NH_3_ measured from tailings at a 0.3-m depth in Till was lower than the concentrations measured in Spray and Trench. This was an unexpected result, as previous studies had reported mixing treatment solutions with soil material provided a homogenous distribution with increasing sample depth (Mujah et al. 2017).

**Table 5:** Ammonia concentrations (mg/L) measured in tailings sample leachate using shake flask extraction method

Depth (m)	NH_3_ concentration (mg/L)			
	Control	Spray	Till	Trench
0	< 0.05	59	63	138
0.3	0.14	21	3.4	31

Results from the water quality analysis show this treatment has the potential to reduce the potential for acid generation by sulfidic tailings. The near-neutral pH maintained may have slowed the incidence of Fe (II) oxidation reactions that contribute to ARD formation. This is supported by lower fractions of sulfate measured in MICP-treated cells relative to the Control. In all cases, the MICP-treated cells exhibited increases in pore water pH over the course of the test period. This increase is likely attributed to the resultant carbonate, ammonium, and hydroxyl ions as products of urea hydrolysis (Choi et al. 2020; Okwadha and Li 2010).

In treated cells, the elevated NH_3_ concentrations observed in the porewater indicated the occurrence of ureolysis and confirm the ureolytic activity of *S. pasteurii* in the tailings environment. It was observed that NH_3_ concentrations peaked after 14 days; then, they began to decrease. There was no statistically significant correlation between viable bacteria and NH_3_ concentrations, indicating that observed decreases NH_3_ within porewater were independent of the decrease in microbial population. This change could be attributed to the volatilization of converted ammonia over the test duration (Kirk and Nye 1991). As the pH of porewater increases, the conversion of NH_4_^+^ (pKa = 9.25) to NH_3_ is more favorable, which increases volatilization potential (Gui et al. 2018). This can also be attributed to a decrease in the availability of urea for ureolysis, as this would ultimately decrease NH_3_ formation (Bhaduri et al. 2016; Lauchnor et al. 2015).

### Future work

While the results show promise for MICP as potential for tailings attenuation, additional research and analysis is required prior to further scale-up. Based on the requirements for this particular test site, a tailings strength measurement of approximately 100 kPa was deemed suitable for proceeding further with closure activities. While the results of this study did not yield this level of material strength, it is believed that further optimization of the spray technique could enable stronger and more evenly cemented tailings. Assessing the behavior of MICP-treated tailings over a longer monitoring period will provide a better understanding of its structural and geochemical behavior and provide insight into the long-term viability of *S. pasteurii* in a tailings environment. Longer-term testing would also be necessary to evaluate the length of time NH_3_ remains in the tailings porewater, as elevated concentrations still remained after the 28-day testing period, and the presence of high concentrations of NH_3_ could have negative environmental implications. Further studies should also assess the robustness of this treatment under more representative climate conditions, i.e., in a field pilot, to assess the effects of seasonal temperatures, humidity, and rainfall patterns on treatment longevity and potential maintenance requirements. Additionally, further a more in-depth analysis of the interactions between *S. pasteurii* and native microbial populations is warranted for better understanding of its impact on MICP.

## Conclusions

This paper examined the feasibility of using MICP for the treatment of coal tailings, and the effectiveness of various large-scale application methods. The spraying of treatment solutions for the Spray method yielded the best overall result as it resulted in an increase in material surface strength and CaCO_3_ with minimal disruption to the material. This technique would be ideal for large-scale applications, as irrigation equipment is widely used, non-intrusive, and enables multiple applications. It is noted that these increases were variable throughout regions of the cell and therefore further optimization of this application method would be necessary. The use of rototilling equipment for the Till treatment provided a homogenous distribution of treatment solution, and increases in CaCO_3_ content; however, the disruption to the tailings material was found to be less effective for facilitating soil cementation. The formation of a shallow trench using the Trench method was found to be an inadequate application method as only marginal improvement to surface strength was observed and a wide variation in measurements indicating resultant CaCO_3_ precipitation was spatially heterogenous. Elevated concentrations of bacterial species present in the tailings suggest *Sporosarcina pasteurii* remains robust to bioaugmentation in scaled applications. No significant declines in the pH of porewater were observed in the tailings over the 28-day monitoring period; however, longer-term studies are recommended in the future to evaluate the impact of MICP treatment on geochemical stability and water quality.

## Data Availability

The datasets used and/or analyzed during the current study are available from the corresponding author on reasonable request.
